# Exoproteomic analysis of two MLST clade 2 strains of *Clostridioides difficile* from Latin America reveal close similarities

**DOI:** 10.1038/s41598-021-92684-0

**Published:** 2021-06-24

**Authors:** Dvison de Melo Pacífico, Cecília Leite Costa, Hercules Moura, John R. Barr, Guilherme Augusto Maia, Vilmar Benetti Filho, Renato Simões Moreira, Glauber Wagner, Regina Maria Cavalcanti Pilotto Domingues, Carlos Quesada-Gómez, Eliane de Oliveira Ferreira, Gerly Anne de Castro Brito

**Affiliations:** 1 Department of Morphology, Faculty of Medicine, Federal University of Ceará, Fortaleza, CE 60430-170 Brazil; 2 Laboratory of Bacteriology, Department of Pathology, Faculty of Medicine, Federal University of Ceará, Fortaleza, CE Brazil; 3 Division of Laboratory Sciences, Centers for Disease Control and Prevention, Atlanta, GA USA; 4 Microbiology, Immunology and Parasitology Department, Laboratory of Bioinformatics, Federal University of Santa Catarina, Florianópolis, SC Brazil; 5 State of Santa Catarina University, Veterinary College, Lages, Brazil; 6 Institute of Microbiology Paulo de Góes, Laboratory of Anaerobic Biology, Federal University of Rio de Janeiro, Rio de Janeiro, Brazil; 7 Facultad de Microbiología and Centro de Investigación en Enfermedades Tropicales, Universidad de Costa Rica, San José, Costa Rica

**Keywords:** Bacteria, Bacterial pathogenesis, Microbiology, Protein analysis, Proteome informatics

## Abstract

*Clostridioides difficile* BI/NAP1/ribotype 027 is an epidemic hypervirulent strain found worldwide, including in Latin America. We examined the genomes and exoproteomes of two multilocus sequence type (MLST) clade 2 *C. difficile* strains considered hypervirulent: ICC-45 (ribotype SLO231/UK[CE]821), isolated in Brazil, and NAP1/027/ST01 (LIBA5756), isolated during a 2010 outbreak in Costa Rica. *C. difficile* isolates were cultured and extracellular proteins were analyzed using high-performance liquid chromatography-tandem mass spectrometry. Genomic analysis revealed that these isolates shared most of the gene composition. Only 83 and 290 NAP1/027 genes were considered singletons in ICC-45 and NAP1/027, respectively. Exoproteome analysis revealed 197 proteins, of which 192 were similar in both strains. Only five proteins were exclusive to the ICC-45 strain. These proteins were involved with catalytic and binding functions and indirectly interacted with proteins related to pathogenicity. Most proteins, including TcdA, TcdB, flagellin subunit, and cell surface protein, were overrepresented in the ICC-45 strain; 14 proteins, including mature S-layer protein, were present in higher proportions in LIBA5756. Data are available via ProteomeXchange with identifier PXD026218. These data show close similarity between the genome and proteins in the supernatant of two strains with hypervirulent features isolated in Latin America and underscore the importance of epidemiological surveillance of the transmission and emergence of new strains.

## Introduction

*Clostridioides difficile* (previously named *Clostridium difficile*), a gram-positive bacillus, spore-forming anaerobic bacterium, is considered the major cause of antibiotic-associated diarrhea in hospitalized patients worldwide^1^. The main virulence factors of *C. difficile* are two potent toxins, TcdA and TcdB. Other proteins related to the observed inflammatory response and colonization in *C. difficile* infection (CDI), including surface layer (S-layer) proteins and flagellin, also have been described^1^. In the early 2000s, the epidemiology of CDI drastically changed with the emergence of BI/NAP1/ribotype 027, an epidemic and hypervirulent strain. This strain caused numerous outbreaks and deaths in North America, Canada, and several countries in Europe^2,3^. More recently, other toxigenic *C. difficile* ribotypes, including 001, 014, and 078^4^, have emerged as main causes of CDI. In Latin America, CDI cases associated with the epidemic strain were reported in Costa Rica, Panama, Chile, and Colombia. A ribotype 027 strain susceptible to fluoroquinolones was detected in Argentina^5^.

Proteomics, the large-scale analysis of proteins, provides information complementary to that obtained through genomics^6–8^. Proteomics allows analysis of proteins related to *C. difficile* antimicrobial resistance, pathogenicity, and metabolic activity. From that analysis, we can better understand molecular mechanisms associated with CDI and potentially can identify cellular targets for therapeutic purposes^6,8^.

Few studies have analyzed the molecular epidemiology of CDI in Latin America. A recent study compared whole-genome sequences of 25 NAP1, RT027, or ST01 *C. difficile* clinical isolates with 129 isolates from the same genotype collected worldwide. These lineages entered Mexico, Costa Rica, Honduras, and Chile from different geographic areas, suggesting that the B1/NAP1/RT027/ST01 isolates from these countries are susceptible to acquiring distinct single-nucleotide polymorphisms and genes implicated in antibiotic resistance^5^. The epidemic strain, NAP1/027, had not been isolated in Brazil to date; however, clade 2 strains have been isolated in two locations, including the one analyzed here.

The aim of this study was to use a proteomic and genomic approach to compare two MLST clade 2 *C. difficile* strains with hypervirulent features, ICC-45 (ST41)^9^ and NAP1/RT027 (ST01)^2,9^, isolated in Brazil and Costa Rica, respectively.

## Results and discussion

### *C. difficile* strains homologous proteins

**Figure 1:**
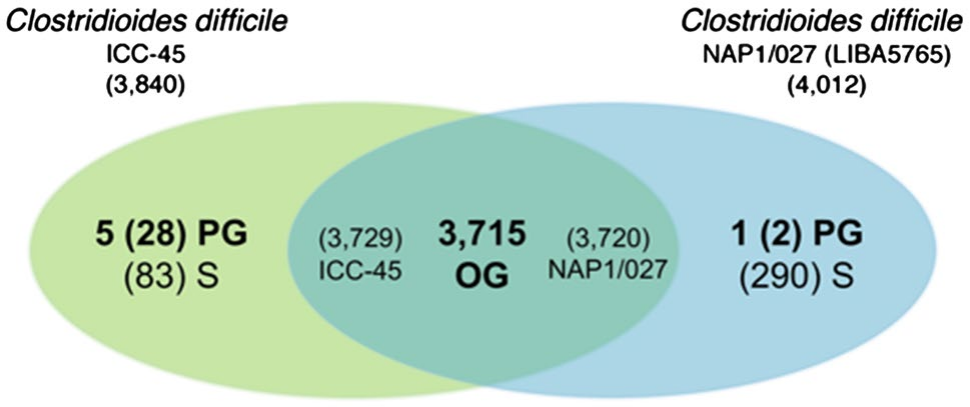
Homology analysis of *Clostridioides difficile* (formerly *Clostridium difficile*) ICC-45 and NAP1/027 (LIBA5756) strains. A total of 3715 orthologous groups (OG) were found between the two strains: 3729 proteins from *C. difficile* ICC-45 and 3720 from *C. difficile* NAP1/027 (LIBA5756). *C. difficile* ICC-45 has 3840 identified proteins, five paralogous groups (PG) with 28 proteins, and 83 singletons (S). *C. difficile* NAP1/027 (LIBA5756) has 4012 identified proteins, one paralogous group (PG) with two proteins, and 290 singletons (S). Numbers inside the parentheses “( )” represents the total number of proteins.

Homology analysis of *C. difficile* ICC-45 revealed a total of 3840 proteins. Of these, 111 were exclusive of this strain. Among those 111 exclusive proteins of *C. difficile* ICC-45, 83 were singletons and 28 were paralogous proteins. *C. difficile* NAP1/027 (LIBA5756) had 4012 proteins identified, of which 292 were exclusive to this strain. Moreover, 290 of these *C. difficile* NAP1/027 exclusive proteins were singletons, and only two were paralogous proteins (Fig. 1).

The numbers of proteins identified in the *C. difficile* strains in this study were similar to the numbers of proteins identified in other strains available on GenBank/NCBI (access codes: NZ_CM000658.1, NZ_CM000637.1, NZ_CM000661.1). Such strains were isolated from patients diagnosed with severe *C. difficile*-associated disease in hospital environments and were analyzed in an unpublished comparative genome study. Two of the three *C. difficile* strains (NZ_CM000658.1 and NZ_CM000661.1) corresponded to NAP1 strains; the other strain (NZ_CM000637.1) was classified as a NAP2-like strain. Of note, one paralogous group composed of 20 copies of the protein “IS200/IS605 family transposase ISBth17” was found in the *C. difficile* ICC-45 genome.

We found that 3729 proteins of *C. difficile* ICC-45 and 3720 proteins of *C. difficile* NAP1/027 (LIBA5756) are included in 3715 orthologous groups (Fig. 1). The reason why *C. difficile* ICC-45 has nine more orthologous proteins than *C. difficile* NAP1/027 (LIBA5756) is that 17 orthologous groups had an uneven number of proteins shared between the two strains (Supplementary Table S1).

We identified that 3689 groups of the 3715 orthologous groups were shared between the two *C. difficile* strains and were composed of only one protein from each strain. Furthermore, 99.9% of the proteins that belong to these shared orthologous groups had a high shared sequence similarity (greater than 95%). This result is corroborated by Costa et al*.*^9^, who showed that both strains had a close phylogenetic relationship and belonged to the same hypervirulent clade (clade 2).

### Analysis of proteins identified in culture supernatants of *C. difficile* strains

A total of 197 proteins were identified in the supernatants of the two *C. difficile* strains. Supplementary Table S2 lists all proteins identified, their accession numbers, and total spectrum counts.

Strains ICC-45 and NAP1/027 (LIBA5756) shared 192 proteins. Five proteins were only detected in the ICC-45 strain: 1) phosphoglyceromutase (gpmI; engaged in the glycolytic pathway), 2) delta-aminolevulinic acid dehydratase (hemB; involved with biosynthesis of porphyrins, metal ligands, and proteasomal activity)^4^, 3) Rrf2 family transcriptional regulator (iscR; a DNA ligand), 4) ribulose phosphate (rpe1; implicated in carbohydrate metabolism), and 5) a hypothetical protein (CD630_1953) (Table 1).

**Table 1:** Proteins secreted exclusively by *Clostridioides difficile* ICC-45 strain.

Identified proteins	Access number (gi)	Gene name^a^	MW (kDa)^a^
Phosphoglyceromutase	gi|255308206	gpmI	56
Hypothetical protein	gi|126699562	CD630_1953	44
Delta-aminolevulinic acid dehydratase	gi|255308448	hemB	36
Rrf2 family transcriptional regulator	gi|126698875	iscR	16
Ribulose-phosphate 3-epimerase	gi|126700194	rpe1	24

^a^Functional annotations were taken from the Swiss protein database (https://www.uniprot.org).

The orthology analysis showed that the coding sequences for these five proteins were present in the genomes of both strains. Gene regulation might explain such a difference in the expression of the five proteins. Dupuy et al.^10^ found that several environmental factors affected protein expression in *C. difficile*. Furthermore, pathogenic organisms have well-regulated control of the expression of their proteins to survive within the host^11,12^.

Considering the ICC-45 exclusive proteins, phosphoglyceromutase (gpmI) plays an essential role in glycolysis and gluconeogenesis. This enzyme interconverts 3-phosphoglyceric acid and 2-phosphoglyceric acid^13^. Nukui et al.^14^ showed that cofactor-independent phosphoglyceromutase is a crucial enzyme for the growth of cells and spores in *Bacillus* species.

Members of the Rrf2 family (transcriptional regulator) are relatively small proteins (12–18 kDa) represented by four regulators (CymR, NsrR, RirA, and IscR)^15^. The protein iron-sulfur biosynthesis regulator (IscR) houses a cluster [2Fe-2S] that coordinates the use of iron and cysteine to form the Fe/S cluster^16^. In *Escherichia coli* and other bacteria, the genes involved in this process are regulated in response to the availability of [Fe-S] through the IscR protein and, consequently, are induced during iron deficiency and oxidative stress^14,15^.

Among the exclusive proteins from the ICC-45 strain, we identified a conserved hypothetical protein of 44 kDa. After comparison in genomic databases, we determined that the protein is 100% identical to coenzyme F_420_: γ-glutamyl ligase (FbiB) in *C. difficile*. Coenzyme F_420_ is a group of active redox cofactors, including FbiB, found mainly in archaea and actinobacteria (including mycobacteria)^17^. Studies have suggested that coenzyme F_420_ protects *Mycobacterium tuberculosis* against oxidative and nitrosative stress during pathogenesis^18,19^.

Regarding the 192 proteins shared among the *C. difficile* strains, 26 were subjectively selected based on the best knowledge of their function and role in the bacteria. Those 26 proteins were categorized by activity into six groups: 1) pathogenicity (toxins, cell surface proteins, flagellar proteins, cell wall proteins, hydrolases and proteases), 2) resistance to antimicrobials (beta-lactamases and pyruvate-ferredoxin), 3) oxidative stress and thermal shock (chaperones), 4) resistance to nitric oxide (nitric oxide reductase flavorubredoxin), 5) metabolism and catalytic activity (trehalose-6-phosphate hydrolase and cysteine desulfurase), and 6) other activities (transcription elongation factor) (Table 2).

**Table 2:** Relative amount of proteins secreted by *Clostridioides difficile* strains ICC-45 and NAP1/027 (LIBA5756).

Identified proteins	Access number (gi)	Gene name^a^	MW (kDa)^a^	Total Spectrum^b^ (ICC-45)	Total Spectrum^b^ (NAP1)
**Proteins involved in pathogenicity**					
Cell surface protein (S-layer precursor protein)	gi|255307831	slpA	76	5359	4001
Toxin A	gi|255305655	tcdA	308	3399	2202
Toxin B	gi|126698238	tcdB	269	2633	1501
Cell surface protein	gi|1001999562	cwp19	77	45	37
Cell wall protein	gi|400927472	cwp22	71	18	4
Cell-wall hydrolase	gi|126700384	CD630_2768	25	30	11
Cell wall binding repeat 2 family protein	gi|531118578	cwp28	51	13	2
Flagellin subunit	gi|260685615	fliC	34	38	0
Cell surface protein (putative cell surface-associated cysteine protease)	gi|260210525	cwp84	87	62	14
**Proteins involved in antimicrobial resistance**					
Beta-lactamase family protein	gi|531115368	QEW_1874	112	49	14
Pyruvate-ferredoxin oxidoreductase	gi|126700296	Pfo	128	895	650
Nitroreductase	gi|126700191	CD630_2572	24	12	8
**Proteins related to oxidative stress and thermal shock**					
Rubrerythrin	gi|126699128	CD630_15240	20	515	573
Cysteine desulfurase	gi|126698876	iscS2	43	115	52
Chaperone DnaK	gi|126700078	dnaK	66	527	335
60 kDa chaperonin	gi|255305190	groL	57	312	167
Heat shock protein 90	gi|126697845	htpG	75	19	3
**Proteins involved in resistance to nitric oxide**					
Anaerobic nitric oxide reductase flavorubredoxin	gi|126698752	norV	44	171	58
Putative nitric oxide reductase flavoprotein	gi|255306635	CDR20291_1521	94	87	84
**Proteins involved with metabolism and catalytic activity**					
Trehalose-6-phosphate hydrolase	gi|126700708	treA	66	266	156
Phosphoenolpyruvate-protein phosphotransferase	gi|260687988	ptsI	63	114	75
Chain D, Alanine Racemase	gi|645985739	alr2	43	43	9
**Proteins involved in other activities**					
d-Proline reductase PrdA	gi|126700863	prdA	67	39	22
Transcription elongation factor GreA	gi|126701179	greA	18	21	1
Cold shock protein CspB	gi|126698954	cspB	7	663	670
Pilin	gi|255308544	CD630_3513	18	32	26

^a^Functional annotations were taken from the Swiss protein database (https://www.uniprot.org).

^b^Concentration based on the amount of total spectrum found using Scaffold (v. 4.8.6.0; Proteome Software Inc., Portland, Oregon, USA).

In comparison with NAP1/027, increased proportions of proteins involved in CDI pathogenesis were detected in the exoproteome of ICC-45, including cell surface protein-S-layer precursor protein, TcdA, TcdB, cell surface protein (Cwp19), cell wall protein (Cwp22), cell-wall hydrolase, cell wall binding protein (Cwp28), flagellin (FliC), and cysteine protease (Cwp84) (Table 2). These proteins contribute to the inflammatory response observed in *C. difficile* pathogenesis^20^, which might explain previous reports of similar increased myeloperoxidase, proinflammatory cytokines, oxidative stress response, tissue nitrite, and epithelial damage in an animal model injected with supernatants of these two strains^9^. However, using western blotting, Costa et al.^9^ showed that ICC-45 releases less toxin than does NAP1/027. This divergence might be a result of different cultivation times being used in the two studies (96 h in the previous study, 24 h in the present study). The present study also did not use supernatant filtration. In addition, the antibodies use in the previous studied were not strain-specific, which could underestimate the level of the variant TcdB produced by ICC-45.

Our finding that ICC-45 has a higher proportion of cell surface protein (S-layer precursor protein) than does NAP1/027 (LIBA5756) is in accord with previous findings that ST1 NAP1 produced a low proportion of the unprocessed precursor^21^. However, NAP1/027 (LIBA5756) expressed a higher proportion of mature S-layer protein, which is formed when the SlpA undergoes proteolytic cleavage by the protease Cwp84. The mature S-layer protein consists of two subunit proteins: a low-molecular-weight complex and high-molecular-weight complex thought to play a role in host cell adhesion^22,23^. Using rabbit anti-sera, Quesada-Gómez et al.^21^ measured levels of SlpA in the exoproteome relative to the corresponding amount in lysates of vegetative cells and reported a low proportion of SlpA in the bacteria-free supernatant of ST1_NAP1. We found that the proportion of S-layer proteins in ICC-45 was even less than that of the ST1_NAP1 used by Quesada-Gómez et al.^21^ Those authors did use different strains (ST1_NAP1 5712 and 6656), and different methodologies. Thus, it is possible that NAP1/027 (LIBA5756) had higher adhesion to host cells or inflammatory capacity than did ICC-45. This remains to be tested.

In the group of antimicrobial resistance-related proteins, ICC-45 produced higher ratios of proteins from the beta-lactamases family, from pyruvate-ferredoxin oxidoreductase, and from nitroreductase than did NAP1/027 (LIBA5756) (Table 2). Chong et al.^24^ also showed high expression of DNA repair proteins, putative nitroreductases, and the ferric uptake regulator (Fur) in strains with reduced susceptibility or resistance to metronidazole, suggesting that these proteins might be involved in metronidazole resistance. As demonstrated previously^9^, ICC-45 was resistant to ceftriaxone and clindamycin, but susceptible to metronidazole, vancomycin, rifampicin, and different from NAP1/027 susceptible to moxifloxacin and levofloxacin. Some groups of antibiotics, such as cephalosporins, clindamycin and fluoroquinolones are associated with increased risk for development of CDI^25,26^. Therefore, resistance to these antibiotics plays an important role in driving the current epidemiological changes of CDI and, consequently, in the appearance of new ribotypes of *C. difficile*^27^.

In comparison with NAP/027 (LIBA5756), the ICC-45 strain produced almost threefold the amount of cysteine desulfurase protein involved in oxidative stress response. The ICC-45 strain also secretes many more heat shock proteins (chaperones). In turn, the NAP/027 (LIBA5756) strain secretes more rubrerythrin than does ICC-45 (Table 2). Rubrerythrin, a protein responsive to oxidative stress, was described initially for its role in protecting strictly anaerobic bacteria from stress. In agreement with our study, a proteomic analysis of *C. difficile* 630 strain showed an increased level of rubrerythrin in response to thermal stress^28^.

The ICC-45 strain produced almost 3 times the amount of the nitric oxide reductase flavorubredoxin that NAP1/027 (LIBA5756) did (Table 2). ICC-45 also produced larger amounts of putative nitric oxide reductase flavoprotein than did NAP1/027 (LIBA5657). These proteins are involved with protection against the effects of nitric oxide^24^. Because nitric oxide is important in the host defense against pathogens, the increased secretion of putative nitric oxide reductase flavoprotein by ICC-45 might contribute to worse CDI outcomes.

In comparison with NAP1/027 (LIBA5756), ICC-45 expressed more trehalose-6-phosphate hydrolase, phosphoenolpyruvate-protein phosphotransferase, and alanine racemase, all of which are involved with metabolism and catalytic activity (Table 2). Collins et al*.*^29^ showed that dietary trehalose plays a role in the dissemination of two epidemic ribotypes of *C. difficile* (RT027 and RT078). They also showed that the introduction of trehalose as a sweetener in the human diet might have played a relevant role in the emergence of these epidemic and hypervirulent strains.

### Exoproteome proteins

**Figure 2:**
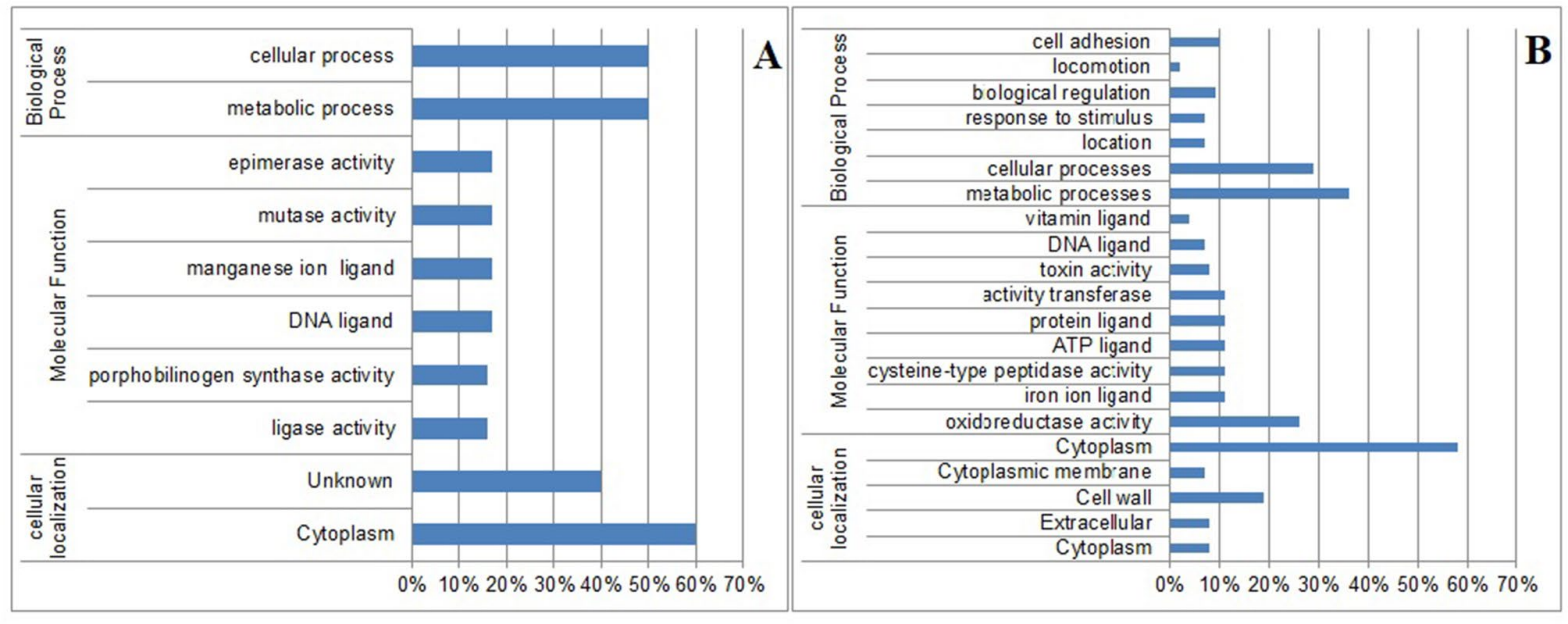
Distribution of proteins according to biological processes, molecular function, and cellular localization. (**A**) Distribution of the exclusive proteins of the ICC-45 strain. (**B**) Distribution of the proteins shared between strains ICC-45 and NAP1/027 (LIBA5756). These *C. difficile* proteins were identified based on the annotations of the ontology gene (GO annotations).

Analysis of the identified proteins using Bast2Go showed that 50% of the ICC-45 strain-exclusive proteins are related to metabolic processes and 50% to cellular processes (Fig. 2A). The 26 selected proteins shared between ICC-45 and NAP1/027 (LIBA5756) are involved in several biological processes, including metabolic processes (36%), cellular processes (29%), cell adhesion (10%), biological regulation (9%), response to stimuli (7%), location (7%), and locomotion (2%) (Fig. 2B). Our data are in accordance with Dresler et al.^30^, who identified a total of 662 proteins, of which more than 120 were involved in metabolic pathways.

The ICC-45 exclusive proteins were categorized by molecular function and activities, including epimerase activity (17%), mutase activity (17%), manganese ion ligand (17%), DNA ligand (17%), porphobilinogen synthase activity (16%), and ligase activity (16%) (Fig. 2A). The 26 shared proteins were distributed in nine functional categories: oxidoreductase activity (26%), transferase activity (11%), ATP ligand (11%), cysteine-type peptidase activity (11%), iron ion ligand (11%), protein ligand (11%), toxin activity (8%), DNA ligand (7%), and vitamin ligand (4%) (Fig. 2B). By contrast, in a proteomic analysis with *C. difficile* strain 630 subjected to thermal stress, of the 447 proteins identified, most were involved in protein synthesis (19.5%) and metabolism of amino acids and molecules (15%)^28^. These divergent results might have resulted from strain 630 being subjected to thermal stress. It then produced more proteins related to protein synthesis and amino acid metabolism. In addition, for our study, selected proteins were analyzed.

Cell localization was analyzed using the PSORTb. Proteins belonging exclusively to the ICC-45 strain were of cytoplasmic origin (60%) or unknown origin (40%) (Fig. 2A). More than half (58%) of the 26 shared proteins also were of cytoplasmic origin. The remainder were localized to the cell wall (19%), extracellular medium (8%), cytoplasmic membrane (7%), or were of unknown origin (8%) (Fig. 2B). Similarly, Jain et al.^28^ showed that 58 of 107 proteins identified in insoluble subproteome of *C. difficile* strain 630 were of cytoplasmic origin. Moura et al.^6^ performed a proteomic analysis of a commercial culture filtrate of *C. difficile* and found that of the 101 proteins identified, the majority (72%) also were of cytoplasmic origin.

### Functional analysis of protein interaction networks

The interactions of the proteins shared between ICC-45 and NAP1/027 (LIBA5756), are remarkably similar in both strains (Supplementary Figure S1A and 1B). The main difference is the five proteins exclusive to the ICC-45 strain (circled in red in Supplementary Figure S1A). Three of these proteins interact with the other ICC-45 proteins that are also found in NAP1/027 (LIBA5756), and two do not present any interactions with the others. The IscR protein, a transcriptional regulator exclusively expressed in ICC-45, interacts directly with the chaperone DnaK protein, which in turn, interacts with the GroL chaperone, and then interacts with pathogenicity proteins (TcdA, TcdB, FliC, Cwp84, and SlpA). Delta-aminolevulinic acid dehydratase (HemB) interacts with ribulose-phosphate 3-epimerase (Rpe1), which then interacts with nitroreductase (CD2572) and pyruvate-ferredoxin oxidoreductase (Pfo) (Supplementary Figure S1A). Pfo also interacts with DnaK and GroL proteins. The DnaK and GroL protein interactions and their links to virulence factors (TcdA, TcdB, FliC, Cwp84, and SlpA) suggest that ICC-45–exclusive proteins might influence the pathogenicity of *C. difficile*. IscR also interacts with cysteine desulfurase (IscS). The interactions of IscR with IscS and with other proteins are involved in cysteine metabolism, being activated under stress conditions or even the absence of iron^16^.

This study documented the substantial similarity of coding sequences of two MLST clade 2 strains of *C. difficile* isolated in Latin America belonging to different pulsotypes and ribotypes. Differences in the expression of specific proteins or in their expressed levels and their interaction with the other proteins might help clarify variations in pathogenicity, antibiotic resistance, metabolism, oxidative stress, resistance to nitric oxide and other aspects of CDI. In a globalized world, the emergence and dissemination of new strains capable of generating outbreaks requires identification of biomarkers and proteins that might lead to better understanding of pathogenesis, treatment, and vaccine development.

## Material and methods

### Strains

Two clade 2 *C. difficile* strains of the Latin America multilocus sequence type (MLST) were used in this study: ICC-45 (ribotype SLO231/UK[CE]821) and NAP1/027/ST01 (LIBA5756). ICC-45 is a toxigenic strain isolated from a 34-year-old patient admitted to the Haroldo Juaçaba Hospital in Fortaleza, Ceará, Brazil, with breast cancer and metastasis to the nervous system, who died from severe diarrhea after 54 days hospitalization. NAP1/027/ST01 (LIBA5756) is a hypervirulent strain isolated in an outbreak in a major Costa Rican hospital and induced a severe clinical presentation in affected patients^2^. Regarding ICC-45, it was classified by pulsed field gel electrophoresis as a new genotype and ribotype classified as ST41 from clade 2 in the MLST. ICC-45 (SL023/UK [CE] 821 is phylogenetically related to the epidemic strain NAP1/027/ST01. Both strains are producers of TcdA, TcdB, and CDT^2,9^. However, unlike the NAP1/027 strain (LIBA 5756), the Brazilian isolate ICC-45 is susceptible to fluoroquinolones and does not have a deletion in the *tcdC* repressor gene for the toxins.

In addition, the TcdA restriction standards of ICC-45 and NAP1 / 027 are identical. For the B1 fragment (catalytic region) of TcdB, however, ICC-45 presented a polymorphism that encodes for a variant TcdB, belonging to the IXb toxinotype, whereas NAP1/027 belongs to the III toxinotype. Like NAP1/027, the ICC-45 is toxigenic (A + B + and CDT+); unlike NAP1/027, it does not have the 18 bp in the 117 position^9^.

The institutional review boards approved these protocols, and written informed consent was obtained from the LARs as deceased patient. All methods were carried out in accordance with the international guidelines for research on humans and principles state in declaration of Helsinki. The ethical approval was obtained by the Ethics and Research Committees of Hospital San Juan de Dios (Costa Rica – protocol CLOBI-SJD-O18-2009) and Hospital Haroldo Juaçaba of the Cancer Institute of Ceará (Brazil – protocol 208.362).

### Gene ortholog *C. difficile* strains

For this study, the genome of the *C. difficile* NAP1/027 (LIBA5756) strain was assembled using the genome data of *C. difficile* ICC-45 strain as a reference (GenBank Assembly Accession GCA_002891495.1). Paired-end reads of *C. difficile* NAP1/027 were obtained from the European Bioinformatics Institute (EBI) database (accession: ERR467583) and their quality was assessed using FastQC (v0.11.8) software. Only reads with a Phred quality score greater than 36 were considered for later analysis. Assembly of paired-end reads was performed using SPAdes (v3.13.1; St. Petersburg State University, Russia) software.

Next, genome annotation of *C. difficile* NAP1/027 (LIBA5756) and *C. difficile* ICC-45 was performed using the Prokka (v1.14.3) software pipeline, allowing for prediction of RNA features with the rnammer and rfam optional parameters. Using the predicted proteins, orthology analysis of *C. difficile* NAP1/027 (LIBA5756) and *C. difficile* ICC-45 was performed using OrthoMCL (v2.0.9) software, using an e-value cut off of 10^−5^. The results obtained by the orthology analysis were treated and reassessed using in-house scripts, considering parameters of similarity and positivity between the protein sequences studied.

### Cell culture and supernatants of the *C. difficile* strains

After growth of the *C. difficile* isolates on *Brucella* agar plates, 2–4 colonies of each were inoculated in 40 mL of brain heart infusion (BHI) broth. The strains were incubated for 24 h at 37 °C in anaerobic jars (90% N_2_, 10% CO_2_, 10% H_2_). After incubation, tubes were centrifuged twice (4000*g*, 8 min, 4 °C) and the supernatants were stored. The supernatants were not filtered. Uninoculated culture media (negative control, BHI broth) were subjected to the same conditions^9^. This experiment was performed in triplicate and protein extracts were used for exoproteomic analysis.

### Precipitation of proteins obtained from *C. difficile* supernatants

All supernatants were precipitated with 1 part chloroform to 4 parts methanol^3^. After the addition of chloroform and methanol, the supernatants were mixed and centrifuged (2000*g*/20 min). The upper phase was discarded, and another 3 volumes of methanol were added. Thereafter, the supernatants were subjected to further washes and the pellets were lyophilized. After drying, precipitates were stored at -80 °C.

Dried supernatants were reconstituted with 500 μL of 25 mM ammonium bicarbonate buffer (pH 8.0) with 1 mM calcium chloride, as described previously^6^. Protein concentrations were determined using a Qubit 2.0 fluorometer (Invitrogen, Waltham, Massachusetts, USA). All tubes were kept at − 80 °C until the moment of use.

### Proteomic analysis: in solution digestion

We mixed 100 mL of each supernatant with 300 μL of AMBIC, then pipetted the mixture into a 30-kDa spin filter (Amicon ULTRA 0.5 mL; MilliporeSigma, Burlington, Massachusetts, USA) for on-filter digestion. Dried peptides were resuspended with 0.1% formic acid and vortexed before separation and mass spectrometry analysis using a Nano-LC (Waters Corporation, Milford, Massachusetts, USA) coupled to an LTQ Orbitrap Elite mass spectrometer (Thermo Fisher, Waltham, Massachusetts, USA)^6^.

### Protein analysis by mass spectrometry

The digestion products, obtained in solution, were analyzed for protein identification using liquid chromatography-tandem mass spectrometry (LC–MS/MS) using an Orbitrap Elite Hybrid Ion Trap-Orbitrap mass spectrometer (Thermo Fischer Scientific, IL, USA) , according to the protocol described previously^6^. The mass spectrometer was operated in a positive mode with a voltage of 35 kV for the acquisition of data based on reading values from 400 to 1400 m/z, at a nominal mass resolution of 60,000 for the acquisition of ions. For the mass spectrometry analysis, the device was programmed to select the 15 most intense ions with two or more loads. The MS/MS acquisition time was 120 min.

### Exoproteins identification and characterization

Protein identification was performed using the Mascot program (v. 2.5.1; Matrix Science, London, UK) with a search for homologous sequences. Mascot was programmed to search for and recognize proteins based on the modified NCBI database using the term "Cdiff-nr-630-base-Sep2015-Vs4," in which the amino acid sequence of ADH 30030 (control) and strains ATCC 43255 and R20291 were concatenated, with trypsin being used as the digestion agent. A peptic mass tolerance of 50 p.p.m. for the parent ion and 0.60 Da for the fragment ions was specified. Deamidation of asparagine and glutamine, oxidation of methionine and cysteine carboxymethyl were also specified in the Mascot as variable modifications, as described by Moura et al.^6^.

The files generated by Mascot were converted for analysis in Scaffold (v. 4.8.6.0; Proteome Software Inc., Portland, Oregon, USA), as previously described^6^. Subsequently, Blast2Go (https://www.blast2go.com/b2ghome), PsortB v. 3.0 (http://www.psort.org/psortb/), and database STRING v. 10.5 (https://string-db.org/) were used to predict the biological functions, subcellular localization of the physical and functional interactions between the identified proteins, respectively.

The mass spectrometry proteomics data have been deposited to the ProteomeXchange Consortium via the PRIDE [1] partner repository with the dataset identifier PXD026218.

## Supplementary Information

Supplementary Figure S1. Protein association in STRING. (**A**) Four functional modules can readily be seen in the network between five proteins exclusive to ICC-45 (red circles), forming tight connected clusters; and 26 selected shared protein from both strains (ICC-45 and NAP1/027). (**B**) Three functional modules are observed in the network with 26 shared proteins in NAP1/027 (LIBA5756) strain.

Supplementary Table S1.

Supplementary Table S2.
