# Supplementary figures and images for: Exoproteomic analysis of two MLST clade 2 strains of Clostridioides difficile from Latin America reveal close similarities
Source: Sci Rep. 2021 Jun 24;11:13273. doi: 10.1038/s41598-021-92684-0 (PMC8225638; doi:10.1038/s41598-021-92684-0)

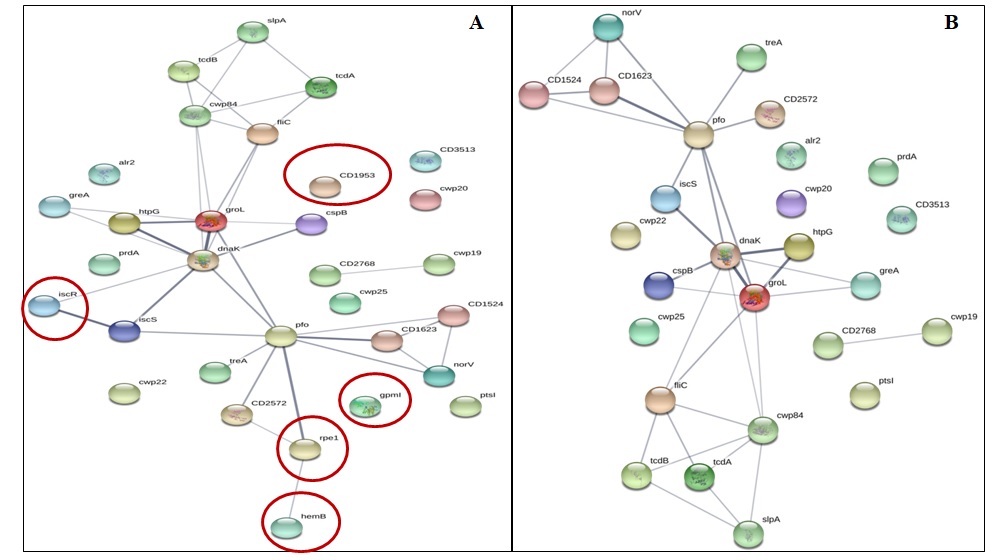

Supplement: Supplementary file 1 — Supplementary Figure S1. Protein association in STRING. (A) Four functional modules can readily be seen in the network between five proteins exclusive to ICC-45 (red circles), forming tight connected clusters; and 26 selected shared protein from both strains (ICC-45 and NAP1/027). (B) Three functional modules are observed in the network with 26 shared proteins in NAP1/027 (LIBA5756) strain. [file 41598_2021_92684_MOESM1_ESM.jpg]
