# Supplementary material for: Exoproteomic analysis of two MLST clade 2 strains of Clostridioides difficile from Latin America reveal close similarities
Source: Sci Rep. 2021 Jun 24;11:13273. doi: 10.1038/s41598-021-92684-0 (PMC8225638; doi:10.1038/s41598-021-92684-0)
Supplement: Supplementary file 2 — Supplementary Table S1. [file 41598_2021_92684_MOESM2_ESM.pdf]

Genomic and exoproteomic analysis of two MLST clade 2 strains of *Clostridioides difficile* from Latin America reveal close similarities

Dvison de Melo Pacífico[1*], Cecília Leite Costa[1,2*], Hercules Moura[3], John R. Barr[3], Guilherme Augusto Maia[4], Vilmar Benetti Filho[4], Renato Simões Moreira[4,5], Glauber Wagner[4], Regina Maria Cavalcanti Pilotto Domingues[6], Carlos Quesada-Gómez[7], Eliane de Oliveira Ferreira[6], Gerly Anne de Castro Brito[1]

Protein	Accession ID	Number of copies	
		ICC-45	NAP1
hypothetical protein	ICC45|MBOGGBJL_00606, ICC45|MBOGGBJL_02577, NAP1|NIKFMFAN_03928, NAP1|NIKFMFAN_04079	2	2
hypothetical protein	ICC45|MBOGGBJL_03996, ICC45|MBOGGBJL_04008, ICC45|MBOGGBJL_04007, NAP1|NIKFMFAN_04061	3	1
IS256 family transposase ISTwi1	NAP1|NIKFMFAN_02317, NAP1|NIKFMFAN_02336, ICC45|MBOGGBJL_03521	1	2
hypothetical protein	NAP1|NIKFMFAN_03620, NAP1|NIKFMFAN_04136, ICC45|MBOGGBJL_01329	1	2
putative protein YqbN	NAP1|NIKFMFAN_03936, NAP1|NIKFMFAN_04016, ICC45|MBOGGBJL_00598	1	2
hypothetical protein	ICC45|MBOGGBJL_00576, ICC45|MBOGGBJL_02556, NAP1|NIKFMFAN_02850	2	1
hypothetical protein	ICC45|MBOGGBJL_00578, ICC45|MBOGGBJL_02558, NAP1|NIKFMFAN_02851	2	1
hypothetical protein	ICC45|MBOGGBJL_00579, ICC45|MBOGGBJL_02559, NAP1|NIKFMFAN_02852	2	1
hypothetical protein	ICC45|MBOGGBJL_00586, ICC45|MBOGGBJL_02562, NAP1|NIKFMFAN_02855	2	1
hypothetical protein	ICC45|MBOGGBJL_00588, ICC45|MBOGGBJL_02565, NAP1|NIKFMFAN_02857	2	1
hypothetical protein	ICC45|MBOGGBJL_01413, ICC45|MBOGGBJL_01414, NAP1|NIKFMFAN_01580	2	1
hypothetical protein	ICC45|MBOGGBJL_01893, ICC45|MBOGGBJL_01894, NAP1|NIKFMFAN_03248	2	1
hypothetical protein	ICC45|MBOGGBJL_02830, ICC45|MBOGGBJL_02833, NAP1|NIKFMFAN_04118	2	1
hypothetical protein	ICC45|MBOGGBJL_04010, ICC45|MBOGGBJL_04011, NAP1|NIKFMFAN_04058	2	1
hypothetical protein	ICC45|MBOGGBJL_00611, ICC45|MBOGGBJL_02582, NAP1|NIKFMFAN_04033	2	1
putative ATP-dependent helicase DinG	ICC45|MBOGGBJL_00407, NAP1|NIKFMFAN_01223, ICC45|MBOGGBJL_00409	2	1

Anaerobic nitric oxide reductase transcription regulator NorR	ICC45|MBOGGBJL_02849, NAP1|NIKFMFAN_02989, NAP1|NIKFMFAN_02988	1	2
	**Total number of proteins**	31	22
