# Supplementary material for: Exoproteomic analysis of two MLST clade 2 strains of Clostridioides difficile from Latin America reveal close similarities
Source: Sci Rep. 2021 Jun 24;11:13273. doi: 10.1038/s41598-021-92684-0 (PMC8225638; doi:10.1038/s41598-021-92684-0)
Supplement: Supplementary file 3 — Supplementary Table S2. [file 41598_2021_92684_MOESM3_ESM.pdf]

Genomic and exoproteomic analysis of two MLST clade 2 strains of *Clostridioides difficile* from Latin America reveal close similarities

Dvison de Melo Pacífico[1*], Cecília Leite Costa[1,2*], Hercules Moura[3], John R. Barr[3], Guilherme Augusto Maia[4], Vilmar Benetti Filho[4], Renato Simões Moreira[4,5], Glauber Wagner[4], Regina Maria Cavalcanti Pilotto Domingues[6], Carlos Quesada-Gómez[7], Eliane de Oliveira Ferreira[6], Gerly Anne de Castro Brito[1]

Number of proteins	Identification of Proteins (*C. difficile*)	Access code	ICC-45 – Total spectrum	NAP1/027 – Total spectrum
1	cell surface protein (S-layer precursor protein	gi|255307831	5359	4001
2	toxin A	gi|255305655	3399	2202
3	toxin B	gi|126698238	2633	1501
4	S-layer protein	gi|126700409	78	2707
5	acetyl-CoA acetyltransferase	gi|126698643	1260	989
6	NAD-specific glutamate dehydrogenase	gi|126697752	826	837
7	pyruvate-ferredoxin oxidoreductase	gi|126700296	895	650
8	aminotransferase	gi|126701292	688	557
9	cold shock protein CspB	gi|126698954	663	670
10	3-hydroxybutyryl-CoA dehydrogenase	gi|126698642	612	410
11	rubrerythrin	gi|126699128	515	573
12	chaperone DnaK	gi|126700078	527	335
13	enolase	gi|126700790	577	357
14	isocaprenoyl-CoA:2-hydroxyisocaproate CoA-transferase	gi|126697964	463	335
15	electron transfer flavoprotein subunit beta	gi|126697969	414	261
16	oxygen-sensitive 2-hydroxyisocaproyl-CoA dehydratase subunit C	gi|126697967	414	255
17	putative surface protein	gi|255308293	291	128
18	electron transfer flavoprotein subunit alpha	gi|126697970	375	253
19	cell surface protein	gi|255307829	286	227
20	formate-tetrahydrofolate ligase	gi|123453210	255	244
21	50S ribosomal protein L7/L12	gi|126697631	361	179
22	gamma-aminobutyrate metabolism dehydratase/isomerase	gi|126699959	220	176
23	aldehyde dehydrogenase family protein	gi|531109986	244	158
24	60 kDa chaperonin	gi|255305190	312	167
25	trehalose-6-phosphate hydrolase	gi|126700708	266	156
26	translation inhibitor endoribonuclease	gi|126700129	266	121
27	peptidase	gi|123452052	276	98
28	peptidase D	gi|126698287	260	168
29	subunit of oxygen-sensitive 2-hydroxyisocaproyl-CoA dehydratase	gi|255305394	225	210
30	aspartate aminotransferase	gi|126698938	234	140
31	glyceraldehyde-3-phosphate dehydrogenase	gi|126700794	185	160
32	D-lactate dehydrogenase	gi|126697963	206	119

33	pyrroline-5-carboxylate reductase	gi|126700900	144	110
34	propanediol utilization phosphotransacylase	gi|126700297	243	89
35	oligopeptide ABC transporter, substrate-binding protein	gi|255307707	145	98
36	phosphoglycerate kinase	gi|126700793	154	76
37	fructose-1,6-bisphosphate aldolase	gi|126697972	136	91
38	alpha/beta hydrolase	gi|126699797	134	58
39	anaerobic nitric oxide reductase flavorubredoxin	gi|126698752	171	58
40	succinate-semialdehyde dehydrogenase	gi|126699960	150	132
41	N-acetylmuramoyl-L-alanine amidase	gi|126700400	120	60
42	toxin A	gi|260685955	2874	1910
43	electron transfer flavoprotein subunit alpha	gi|126698640	149	112
44	electron transfer flavoprotein subunit beta	gi|260686277	105	81
45	ferritin	gi|126699811	119	86
46	phosphoenolpyruvate-protein phosphotransferase	gi|260687988	114	75
47	elongation factor G	gi|126697637	114	49
48	NAD-dependent 4-hydroxybutyrate dehydrogenase	gi|255307361	107	93
49	putative nitric oxide reductase flavoprotein	gi|255306635	87	84
50	rubrerythrin	gi|126699078	324	353
51	glutamyl-aminopeptidase	gi|126699768	91	62
52	cysteine desulfurase	gi|126698876	115	52
53	glucose-6-phosphate isomerase	gi|126700904	101	90
54	proline racemase	gi|126700857	75	94
55	putative cell wall hydrolase	gi|123452506	104	47
56	ABC transporter, substrate-binding lipoprotein	gi|255305875	109	21
57	50S ribosomal protein L1	gi|126697629	83	99
58	butyryl-CoA dehydrogenase	gi|260686276	46	77
59	formate acetyltransferase	gi|255305763	42	30
60	cell surface protein	gi|1001999562	45	37
61	hypothetical protein CD630_16600	gi|126699264	89	52
62	3-hydroxybutyryl-CoA dehydratase	gi|126698641	73	69
63	ferredoxin/flavodoxin oxidoreductase subunit gamma	gi|126697690	75	55
64	M24 family peptidase	gi|126700227	81	53
65	elongation factor Ts	gi|126699756	64	16
66	hypothetical protein CdifA_19096	gi|255308707	45	40
67	acyl-CoA dehydrogenase	gi|126697968	76	51
68	inosine 5-monophosphate dehydrogenase	gi|126699953	59	33
69	beta-lactamase family protein	gi|531115368	49	14

70	NifU-like protein	gi|255306237	45	16
71	manganese-dependent inorganic pyrophosphatase	gi|126697905	66	35
72	cell surface protein (putative cell surface-associated cysteine protease)	gi|260210525	62	14
73	O-acetyl-serine thiol-lyase A	gi|126699198	58	39
74	trigger fator	gi|260688508	70	38
75	glycine cleavage system protein H	gi|126698309	67	57
76	transketolase	gi|126699939	47	21
77	chain D, Alanine Racemase	gi|645985739	43	9
78	elongation factor Tu	gi|126697638	54	31
79	oligopeptide family ABC transporter substrate-binding protein	gi|126698435	53	15
80	rubrerythrin	gi|126698405	37	58
81	2-hydroxyacyl-CoA dehydratase	gi|126699356	47	23
82	6-phosphofructokinase	gi|126701018	57	18
83	formate acetyltransferase	gi|260688484	30	28
84	50S ribosomal protein L10	gi|126697630	41	70
85	tellurium resistance protein	gi|126699238	44	45
86	pyruvate kinase	gi|126701017	43	14
87	D-proline reductase PrdA	gi|126700863	39	22
88	putative aminotransferase	gi|123453053	36	11
89	polynucleotide phosphorylase/polyadenylase	gi|126698917	41	23
90	hypothetical protein CdifA_05660	gi|255306059	33	12
91	ribosome recycling fator	gi|126699754	26	52
92	putative phosphate butyryltransferase	gi|123453214	53	11
93	50S ribosomal protein L5	gi|126697652	19	36
94	putative amino acid racemase	gi|255307859	61	4
95	decarboxylase	gi|260687271	46	46
96	butyrate kinase	gi|126697685	28	30
97	30S ribosomal protein S16	gi|123453547	41	41
98	DNA-directed RNA polymerase subunit beta	gi|126697633	36	7
99	ferredoxin-NADP(+) reductase subunit alpha	gi|260686749	26	25
100	hypothetical protein CdifA_19448	gi|255308768	49	31
101	triosephosphate isomerase	gi|255308207	58	18
102	tellurium resistance protein	gi|126699239	50	52
103	glycine reductase complex component C subunit beta	gi|126699967	14	11
104	adenylate kinase	gi|260685465	41	25
105	50S ribosomal protein L3	gi|126697640	61	2
106	toxin B	gi|260685953	1062	657

107	peptidase	gi|260688699	43	11
108	NUDIX family hydrolase	gi|260686089	32	28
109	hypothetical protein CD630_24460	gi|400927444	26	17
110	putative bifunctional carbon monoxide dehydrogenase/acetyl-CoA synthase	gi|255305718	16	12
111	indolepyruvate oxidoreductase subunit	gi|255307406	22	6
112	bifunctional carbon monoxide dehydrogenase/acetyl-CoA synthase complex subunit alpha/beta	gi|126698308	34	12
113	30S ribosomal protein S8	gi|126697654	34	29
114	cysteinyl-tRNA synthetase	gi|260685418	27	11
115	cell wall protein	gi|400927472	18	4
116	acetyl-CoA decarbonylase/synthase complex subunit gamma	gi|255305729	12	25
117	pilin	gi|255308544	32	26
118	oxidoreductase	gi|126699141	27	12
119	nitroreductase-family protein	gi|126698718	27	6
120	serine hydroxymethyltransferase	gi|126700341	15	1
121	chaperonin GroES	gi|126697766	21	42
122	translation initiation factor IF-3	gi|126698264	30	19
123	hypothetical protein CDR20291_3527	gi|260688868	35	9
124	phosphate butyryltransferase	gi|126697684	35	4
125	thiamine-phosphate synthase	gi|126699205	19	19
126	pyochelin synthetase F	gi|255305434	9	4
127	DNA-directed RNA polymerase subunit alpha	gi|126697669	31	12
128	flagellin subunit	gi|260685615	38	0
129	tellurium resistance protein	gi|126699408	31	9
130	bacteriocin-associated integral membrane family protein	gi|531109313	14	11
131	cell surface protein (S-layer precursor protein)	gi|123451639	77	97
132	hypothetical protein CD630_08070	gi|126698387	22	25
133	hypothetical protein CD630_35710	gi|126701198	24	22
134	diguanylate kinase signaling protein	gi|126699025	32	0
135	30S ribosomal protein S3	gi|126697646	21	19
136	50S ribosomal protein L4	gi|760237126	32	7
137	threonine--tRNA ligase	gi|531119503	11	7
138	50S ribosomal protein L11	gi|126697628	9	13
139	cell-wall hydrolase	gi|126700384	30	11
140	4Fe-4S binding domain-containing protein	gi|126697687	35	5
141	DNA/RNA helicase	gi|400927363	6	2
142	transcription elongation factor GreA	gi|126701179	21	1

143	pyruvate phosphate dikinase	gi|260687649	10	1
144	peptidase T	gi|126698630	13	3
145	adenylosuccinate synthetase	gi|126701282	15	9
146	4-aminobutyrate aminotransferase	gi|255307170	14	2
147	elongation factor P	gi|126698842	14	9
148	heat shock protein 90	gi|126697845	19	3
149	rubredoxin oxidoreductase	gi|260686125	10	12
150	ferredoxin/flavodoxin oxidoreductase subunit alpha	gi|126697688	6	13
151	carbon monoxide dehydrogenase/acetyl-CoA synthase complex, dihydrolipoyl dehydrogenase subunit	gi|260686019	16	0
152	GMP synthase	gi|126697769	12	0
153	1-phosphofructokinase	gi|531111417	20	8
154	n-acetylmuramoyl-l-alanine amidase	gi|260686827	19	5
155	cell division protein FtsZ	gi|126700260	22	0
156	heat shock protein	gi|123451155	9	8
157	tellurium resistance protein	gi|123450620	14	5
158	proline reductase	gi|260688442	13	0
159	cell wall binding repeat 2 family protein	gi|531118578	13	2
160	Nitroreductase	gi|126700191	12	8
161	S-adenosylmethionine synthetase	gi|126697702	13	13
162	Phosphoglyceromutase	gi|255308206	24	0
163	sugar family ABC transporter substrate-binding protein	gi|126700259	12	3
164	putative polysaccharide deacetylase	gi|123452999	21	0
165	DNA polymerase III subunit beta	gi|123452711	14	7
166	Translaldolase	gi|126699947	14	9
167	iron-only hydrogenase, catalytic subunit	gi|260688570	18	0
168	hypothetical protein CdifA_11162	gi|255307139	9	3
169	Xaa-Pro dipeptidase	gi|126699965	12	0
170	30S ribosomal protein S13	gi|126697666	10	4
171	50S ribosomal protein L24	gi|400927242	9	8
172	Oxidoreductase	gi|126700638	10	1
173	aspartate aminotransferase	gi|760239936	11	1
174	putative signaling protein	gi|255305513	3	0
175	hypothetical protein CD630_19530	gi|126699562	17	0
176	sigma-54 factor interaction domain-containing protein	gi|255305861	3	2
177	cell surface protein	gi|760239296	9	2
178	hypothetical protein CD196_1925	gi|260683661	13	0

179	Thioredoxin	gi|126699296	4	5
180	dihydrodipicolinate synthase 3	gi|126700845	5	1
181	50S ribosomal protein L21	gi|126698756	12	8
182	30S ribosomal protein S4	gi|126697668	7	1
183	delta-aminolevulinic acid dehydratase	gi|255308448	14	0
184	phosphohexomutase	gi|126699936	6	0
185	hypothetical protein CdifA_02508	gi|255305433	1	2
186	hemagglutinin/adhesin	gi|126698095	0	2
187	hydrolase beta-lactamase-like	gi|126700357	11	0
188	30S ribosomal protein S7	gi|126697636	5	3
189	Rrf2 family transcriptional regulator	gi|126698875	10	0
190	threonine dehydratase II	gi|126700130	5	0
191	hypothetical protein CD630_19700	gi|126699579	5	1
192	ribulose-phosphate 3-epimerase	gi|126700194	8	0
193	deoxyguanosinetriphosphate triphosphohydrolase-like protein	gi|255306687	6	0
194	DNA primase	gi|260686668	2	1
195	putative hydrolase	gi|123450945	4	0
196	hypothetical protein CD630_02790	gi|126697851	8	0
197	1-(5-phosphoribosyl)-5-[(5-phosphoribosylamino)methyliden eamino] imidazole-4-carboxamide isomerase	gi|260686765	5	1

* Red color: Proteins in greater quantity in NAP1 strain than ICC-45.
